# A phenome-wide association study of genetically mimicked statins

**DOI:** 10.1186/s12916-021-02013-5

**Published:** 2021-06-30

**Authors:** Shun Li, C. M. Schooling

**Affiliations:** 1 School of Public Health, Li Ka Shing Faculty of Medicine, The University of Hong Kong, 7 Sassoon Rd, Pokfulam, Hong Kong, Special Administrative Region of China; 2 School of Public Health and Health Policy, The City University of New York, 55 W 125 St, New York, NY 10027 USA

**Keywords:** Statins, Phenome-wide association study, Calcium, Sex-specific analysis

## Abstract

**Background:**

Beyond their success in cardiovascular disease prevention, statins are increasingly recognized to have sex-specific pleiotropic effects. To gain additional insight, we characterized associations of genetically mimicked statins across the phenotype sex-specifically. We also assessed whether any apparently non-lipid effects identified extended to genetically mimicking other widely used lipid modifiers (proprotein convertase subtilisin/kexin type 9 (PCSK9) inhibitors and ezetimibe) or were a consequence of low-density lipoprotein cholesterol (LDL-c).

**Methods:**

We performed a sex-specific phenome-wide association study assessing the association of genetic variants in *HMGCR*, mimicking statins, with 1701 phenotypes. We used Mendelian randomization (MR) to assess if any non-lipid effects found were evident for genetically mimicked PCSK9 inhibitors and ezetimibe or for LDL-c.

**Results:**

As expected, genetically mimicking statins was inversely associated with LDL-c, apolipoprotein B (ApoB), and total cholesterol (TC) and positively associated with glycated hemoglobin (HbA1c) and was related to body composition. Genetically mimicking statins was also inversely associated with serum calcium, sex hormone-binding globulin (SHBG), and platelet count and positively associated with basal metabolic rate (BMR) and mean platelet volume. Stronger associations with genetically mimicked statins were evident for women than men for lipid traits (LDL-c, ApoB, and TC), calcium, and SHBG, but not for platelet attributes, body composition, or BMR. Genetically mimicking PCSK9 inhibitors or ezetimibe was also associated with lower lipids, but was not related to calcium, SHBG, BMR, or body composition. Genetically higher LDL-c increased lipids and decreased BMR, but did not affect calcium, HbA1c, platelet attributes, or SHBG with minor effects on body composition.

**Conclusions:**

Similar inverse associations were found for genetically mimicking statins on lipid traits in men and women as for other lipid modifiers. Besides the positive associations with HbA1c, BMI (which may explain the higher BMR), and aspects of body composition in men and women, genetically mimicking statins was additionally associated with platelet attributes in both sexes and was inversely associated with serum calcium and SHBG in women. This genetic evidence suggests potential pathways that contribute to the effects of statins particularly in women. Further investigation is needed to confirm these findings and their implications for clinical practice.

**Supplementary Information:**

The online version contains supplementary material available at 10.1186/s12916-021-02013-5.

## Background

Statins, 3-hydroxy-3-methyl-glutaryl coenzyme A reductase (HMGCR) inhibitors, reduce low-density lipoprotein cholesterol (LDL-c) significantly, resulting in a commeasurable reduction in morbidity and mortality from cardiovascular disease (CVD) [1, 2]. Beyond their effectiveness as a cardiovascular intervention via lipid modification, pleiotropic effects of statins have long been suggested [3–5]. Cardiovascular-related pleiotropic effects of statins may include beneficial effects via a range of potentially inter-related factors, including inflammatory responses [6, 7], endothelial function [5], possibly prothrombin time, and sex hormones [8]. More recently, Mendelian randomization (MR) studies have indicated that statins may reduce the risk of cancer by a lipid-independent pathway [9] as well as specifically reducing epithelial ovarian cancer [10]. Taken together, these studies highlight potential sex differences in the mechanisms underlying statins’ protective effects on CVD and overall mortality. However, exactly what mechanisms underlie statins’ complex sex-specific effects and how they might affect the positioning of statins in disease prevention and treatment remains unclear. Randomized trials are rarely designed or powered to investigate mechanisms or pleiotropic effects, although effects of statins on body weight and diabetes have been identified from a meta-analysis of trials [11, 12].

To identify statins’ pleiotropic effects, previous studies have used genetically mimicked statins to assess statins’ metabolomic profile [13], compared it with that of proprotein convertase subtilisin/kexin type 9 (PCSK9) inhibitors [14], and compared statin’s lipoprotein signature with that of cholesteryl ester transfer protein (CETP) inhibitors [15]. However, none of these studies has been comprehensive across the phenotype or sex-specific, when differences by sex are evident for the incidence of CVD [16] and some cancers [17, 18], highlighting the possibility of sex-specific pathways and sex-specific impacts. To fill this gap, we conducted a phenome-wide association study (PheWAS), a summary statistics-based [19] genotype-to-phenotype approach [20, 21], to assess systematically the sex-specific associations of genetically mimicking statins with a wide range of conditions and related phenotypes, using the largest available genome-wide association studies (GWAS), with validation where possible. The use of genetic mimics largely avoids confounding owing to the random allocation of alleles at conception [22]. To assess if any pleiotropic effects identified from the PheWAS were unique to statins, we also assessed whether these pleiotropic effects were evident for (a) genetically mimicking the major lipid modifiers in common use, i.e., PCSK9 inhibitors and ezetimibe, and (b) LDL-c.

## Methods

### Genetic mimics of statins, and other lipid modifiers

A well-established genetic mimic of statins is the T allele of rs12916 in the *HMGCR* gene [13]. rs12916 downregulates hepatic HMGCR expression and hence lowers serum LDL-c [12]. We used the lead SNPs rs11591147 [23] and rs10260606 [8, 24] to genetically mimic PCSK9 inhibitors and ezetimibe respectively (Additional file 1: Table S1). In sensitivity analysis for significant phenotypes for rs12916, we used all genetic variants mimicking statins taking into account their correlations, and similarly for PCSK9 inhibitors and ezetimibe (correlation coefficient matrixes shown at Additional file 1: Table S2-4). Finally, as previously, we used 56 independent single nucleotide polymorphisms (SNPs) from different genomic regions (r^2^ < 0.01) to genetically predict LDL-c (Additional file 1: Table S5) [25].

### Phenotype data sources

To date, few GWAS have focused on sex-specific analysis. Sex-specific summary genetic associations for many diseases and phenotypes are available from the UK Biobank study of 502,665 people recruited in 2006 to 2010 from Great Britain intended to be aged 40 to 69 years [26]. The UK Biobank study involved a comprehensive baseline assessment including questionnaire, interview assessment, physical measures, and sample collection. Follow-up of the participants for health-related outcomes by record linkage to hospital and death records is ongoing [26]. Here, we used the UK Biobank to perform the primary analysis and replicated the results using other publicly available consortia data, where possible.

Currently, UK Biobank summary statistics curated in the MRC Integrative Epidemiology Unit (IEU) OpenGWAS database [27] include 3948 phenotypes including diagnoses, current health status, treatment records, biochemical assays, anthropometrics, physical measurements, family history, lifestyle, and psychological health, with detailed information on their derivation (source, original questionnaire, or measurement) available on the UK Biobank website (https://biobank.ndph.ox.ac.uk/showcase/search.cgi) keyed on the phenotype or ID (Additional file 1: Table S6). Sex-specific summary statistics for the UK Biobank were provided by Neale Lab (http://www.nealelab.is/uk-biobank/) based on up to 361194 white-British people.

### Phenotype categorization

Binary outcomes were considered in groups, corresponding to selected International Classification of Diseases (ICD)-9/10 chapters, i.e., circulatory, endocrine, respiratory, neoplasms, digestive, neurological, musculoskeletal, gynecologic and obstetric, hematopoietic, dermatologic, genitourinary, mental health, infectious diseases, sense organs, injuries and poisonings, symptoms, and others. Categorical and ordered phenotypes were classified in groups using the original UK Biobank categories (https://biobank.ndph.ox.ac.uk/showcase/cats.cgi), i.e., blood count, blood biochemistry, and physical measures.

### Inclusion and exclusion criteria

**Fig. 1:**
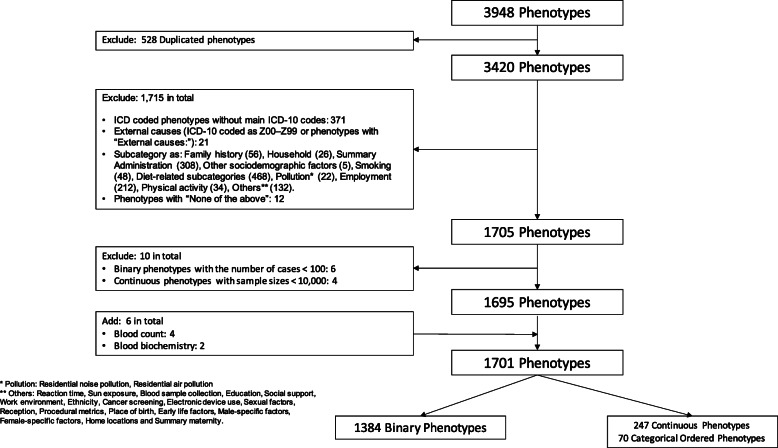
Flow chart of phenotype inclusion for the PheWAS of genetically mimicking statins

Duplicate phenotypes were excluded. To ensure power, binary phenotypes with less than 100 cases and continuous or categorical ordered phenotypes with a sample size less than 10,000 were excluded [28]. ICD-coded binary phenotypes without main ICD codes or with external causes (codes as Z00–Z99) were also excluded. The original categories provided by the UK Biobank study (https://biobank.ndph.ox.ac.uk/showcase/cats.cgi) were used. Subcategories unlikely to reflect effects of statins, such as the subcategory covering family history, hospital administration information, household attributes, lifestyle (smoking, diet, physical activity), environmental exposures (pollutant and sun exposure), employment, socio-demographic factors (education, ethnicity), early life factors, maternity experiences, or other factors unrelated to health status, were excluded. Figure 1 shows the selection of phenotypes with detailed lists of the subcategories excluded.

### Statistical analysis

MR was used to obtain sex-specific effects of genetically mimicking lipid modifiers and of genetically predicted LDL-c scaled to a standard deviation (SD) unit difference in LDL-c [13]. MR estimates were based on Wald estimates (SNP on outcome divided by SNP on exposure) which were meta-analyzed using inverse-variance weighting (IVW) with multiplicative random effects when 3 or more SNPs were available, taking any correlations between SNPs into account obtained from the correlation coefficient matrix of corresponding SNPs. Estimates for binary traits extracted from GWAS using linear regression were converted to odds ratios (OR), for presentation, as necessary, using an established approximation [29]. MR-Egger was used as a sensitivity analysis for LDL-c because it can detect violations of the exclusion restriction assumption and provide a robust estimate when the genetic variants have pleiotropic effects on outcomes [30]. Differences by sex were assessed using a two-sided z-test [31]. The level of statistical significance was adjusted for multiple comparisons using a Bonferroni correction: P = 0.05/Np, where Np is the number of phenotypes tested [32], giving a corrected P value of 2.9 × 10^−5^ (0.05/1701).

### Replication

Where possible, associations found in the UK Biobank were validated using appropriate consortia. A two-sided z-test was used to compare the primary and replication results.

Statistical analyses were performed using R (version 4.0.3; R Foundation for Statistical Computing, Vienna, Austria). Associations of selected SNPs with available outcomes were extracted using the TwoSampleMR R package (version 0.5.5). MR analysis was performed using the MendelianRandomization R package (version 0.4.2). Ethical approval is not necessary because this study was conducted using publicly available summary statistics.

## Results

In total, 1384 binary, 70 categorical ordered, and 247 continuous phenotypes were included (Additional file 1: Table S6). Of the 1384 binary phenotypes considered, 135 were circulatory, 55 endocrine, 80 respiratory, 69 neoplasms, 143 digestive, and the remaining 902 were neurological, musculoskeletal, gynecologic and obstetric, hematopoietic, dermatologic, genitourinary, mental health, infectious diseases, sense organs, injuries and poisonings, description of symptoms, and others.

### Sex-specific PheWAS of genetically mimicked statins

A Manhattan plot of rs12916 (Additional file 2: Figure I) shows −log_10_ transformed P-values of all phenotypes included by category. Rs12916 had most associations in the endocrine category for binary phenotypes, and in the blood count, blood biochemistry, and physical measures for continuous phenotypes. Correspondingly, the 49 associations found for rs12916 were mainly with endocrine factors, blood counts, blood biochemistry, and anthropometrics (Additional file 1: Table S7).

For the binary phenotypes considered, the rs12916 T allele was mainly associated with a lower risk of high cholesterol and less use of cholesterol-lowering drugs, including atorvastatin, ezetimibe, and simvastatin. The rs12916 T allele was also negatively associated with snoring. These associations were largely similar by sex.

For the continuous phenotypes considered, the rs12916 T allele was associated with lower levels of several lipid traits including LDL-c, apolipoprotein B (ApoB), and total cholesterol, with stronger associations in women than men. The rs12916 T allele was associated with higher glycated hemoglobin (HbA1c), mean platelet volume (MPV), and BMR, and lower values of some other platelet attributes including platelet count and platelet crit, with similar associations by sex. In addition, the rs12916 T allele was also associated with lower serum calcium and lower sex hormone-binding globulin (SHBG) particularly in women (Table 1). The rs12916 T allele was also associated with a wide range of related anthropometric measures, shown in Table 1 for anthropometric measures (higher weight, body mass index (BMI), hip circumference, and waist circumference) and Additional file 1: Table S8 for body composition measures (higher fat percentage, fat mass, and fat-free mass of the arms, legs, trunk, and whole body; higher levels of impedance of the arms, legs, and whole body; and higher basal metabolic rate). These associations were largely similar by sex. Sensitivity analysis also showed consistent directions and similar effect sizes for the lead SNP or using all 6 SNPs with a correlation matrix for statins (Additional file 1: Table S11 and Additional file 2: Figure II).

**Table 1:** Sex-specific associations of genetically mimicked statins, PCSK9 inhibitors, and ezetimibe (based on rs12916, rs11591147, and rs10260606, respectively) with continuous phenotypes strongly associated with rs12916 (excluding some body composition traits)

Trait	Genetically mimicked statins (rs12916)							Genetically mimicked PCSK9 inhibitors (rs11591147)							Genetically mimicked ezetimibe (rs10260606)						
	Both sexes		Men		Women		P value^**^	Both sexes		Male		Women		P value^**^	Both sexes		Male		Women		P value^**^
	Beta^*^	Pval	Beta^*^	Pval	Beta^*^	Pval		Beta^*^	Pval	Beta^*^	Pval	Beta^*^	Pval		Beta^*^	Pval	Beta^*^	Pval	Beta^*^	Pval	
ApoB	−0.89	1.02 × 10^−112^	−0.88	4.03 × 10^−38^	−0.89	2.49 × 10^−79^	0.001	−0.99	9.88 × 10^−324^	−1.01	5.71 × 10^−151^	−0.97	7.14 × 10^−176^	0.84	−0.90	5.47 × 10^−25^	−0.87	1.70 × 10^−8^	−0.91	4.43 × 10^−19^	0.06
LDL-c	−1.00	4.32 × 10^−144^	−1.00	2.45 × 10^−49^	−1.00	3.25 × 10^−100^	<0.001	−1.00	0	−1.00	1.18 × 10^−150^	−1.00	5.02 × 10^−189^	0.55	−1.00	4.33 × 10^−31^	−1.00	6.92 × 10^−11^	−1.00	8.49 × 10^−23^	0.07
TC	−0.95	9.66 × 10^−134^	−0.97	1.67 × 10^−46^	−0.95	1.40 × 10^−92^	0.002	−0.90	6.07 × 10^−278^	−0.91	3.18 × 10^−124^	−0.90	1.19 × 10^−157^	0.63	−0.90	2.53 × 10^−26^	−0.91	3.16 × 10^−9^	−0.91	1.72 × 10^−19^	0.09
HbA1c	0.19	4.30 × 10^−7^	0.20	2.65 × 10^−03^	0.19	3.66 × 10^−05^	0.61	−0.06	0.013	−0.10	0.011	−0.03	0.329	0.21	0.47	3.12 × 10^−8^	0.41	0.007	0.51	2.32 × 10^−7^	0.15
Calcium	−0.22	1.27 × 10^−7^	−0.15	0.039	−0.26	1.64 × 10^−07^	0.04	−0.01	0.755	−0.02	0.560	0.003	0.935	0.63	0.08	0.401	0.27	0.090	−0.04	0.705	0.13
SHBG	−0.17	5.68 × 10^−6^	−0.09	0.189	−0.25	1.15 × 10^−06^	0.01	0.003	0.893	0.05	0.204	−0.03	0.401	0.14	0.01	0.954	0.06	0.685	−0.05	0.678	0.56
BMR	0.19	5.30 × 10^−19^	0.26	1.01 × 10^−4^	0.29	7.01 × 10^−10^	0.17	−0.01	0.977	−0.03	0.395	0.02	0.637	0.35	−0.02	0.661	0.23	0.120	−0.09	0.367	0.08
MPV	0.21	1.94 × 10^−07^	0.26	9.97 × 10^−5^	0.17	5.25 × 10^−4^	0.62	−0.05	0.068	−0.09	0.022	−0.01	0.746	0.14	−0.44	6.21 × 10^−7^	−0.71	3.70 × 10^−6^	−0.26	0.013	0.09
Platelet count	−0.26	7.03 × 10^−12^	−0.37	3.34 × 10^−8^	−0.20	1.89 × 10^−5^	0.26	0.05	0.040	0.07	0.071	0.04	0.267	0.58	0.21	0.014	0.32	0.039	0.16	0.128	0.64
Platelet crit	−0.18	1.27 × 10^−6^	−0.28	4.30 × 10^−5^	−0.14	4.17 × 10^−3^	0.30	0.03	0.152	0.03	0.471	0.04	0.216	0.75	0.01	0.921	−0.04	0.806	0.02	0.865	0.77
BMI	0.32	5.20 × 10^−24^	0.33	6.11 × 10^−7^	0.31	3.73 × 10^−11^	0.41	0.02	0.894	0.03	0.418	−0.02	0.636	0.36	0.05	0.587	0.15	0.317	−0.14	0.178	0.10
Weight	0.27	1.40 × 10^−22^	0.31	3.07 × 10^−6^	0.30	1.15 × 10^−10^	0.33	0.01	0.913	0.03	0.460	−0.03	0.406	0.27	0.01	0.915	0.20	0.198	−0.10	0.321	0.11
HC	0.31	1.30 × 10^−21^	0.35	1.90 × 10^−7^	0.28	5.20 × 10^−9^	0.88	0.02	0.542	0.01	0.768	0.02	0.535	0.84	0.08	0.366	0.09	0.545	−0.15	0.135	0.14
WC	0.24	2.10 × 10^−16^	0.29	1.71 × 10^−5^	0.26	2.33 × 10^−8^	0.52	0.05	0.088	0.01	0.764	0.06	0.067	0.31	0.02	0.773	0.07	0.657	−0.05	0.610	0.50

^*^Relative effect on LDL-c, i.e., the sex-specific effect size for each trait divided by the absolute value of the corresponding sex-specific effect size for LDL-c

^**^P value for Z test

*HC* hip circumference, *WC* waist circumference

#### Associations for PCSK9 and ezetimibe

Of the 49 associations found for rs12916, only the associations with lipids were evident for PCSK9 inhibitors (based on the rs11591147 T allele), while associations with lower lipids, higher HbA1c, and lower MPV were evident for ezetimibe (based on the rs10260606 Gallele), as shown in Table 1. However, other associations were not evident for either PCSK9 inhibitors or ezetimibe, as shown in Table 1 and Additional file 1: Table S8. Sensitivity analysis also showed consistent directions and similar effect sizes for the lead SNPs or using alternative SNPs with correlation matrixes for PCSK9 inhibitors and ezetimibe (Additional file 1: Table S12-13 and Additional file 2: Figure III and IV).

#### Associations for LDL-c

Of the 6 associations with binary phenotypes found for rs12916 (Additional file 1: Table S9), genetically predicted LDL-c was associated with higher self-reported high cholesterol and four cholesterol-lowering treatment phenotypes, but not with snoring, where rs12916 was more strongly associated with ezetimibe use in men than women for (*P* value = 0.003). Of the 43 associations with continuous phenotypes, lipid and body composition traits were a consequence of genetically predicted LDL-c. Specifically, LDL-c was positively associated with ApoB and total cholesterol. LDL-c was also and negatively associated with BMR and with most anthropometric and body composition traits with much smaller magnitude of effects than for statins. LDL-c was not associated with HbA1c, calcium, SHBG, platelet count, platelet crit, or MPV (*P* values > 0.05). Here, the univariable MR-Egger intercepts for LDL-c on ApoB and waist circumference were significant in both sexes (*P* = 0.022, 0.038, respectively), suggesting that the IVW estimate was invalid due to potential pleiotropic effects. The MR-Egger estimates for the association of LDL-c with ApoB and waist circumference were similar to the IVW estimates. These associations with continuous phenotypes were generally similar by sex, as shown in Table 2 and Additional file 1: Table S9.

**Table 2:** Sex-specific MR estimates of genetically predicted LDL-c on continuous phenotypes strongly associated with rs12916 (excluding some body composition traits)

Trait	IVW									MR-Egger											
	Both sexes			Men			Women			Both sexes				Men				Women			
	Beta	95% CI	P value	Beta	95% CI	P value	Beta	95% CI	P value	Beta	95% CI	P value	*P*_Intercept_	Beta	95% CI	P value	*P*_Intercept_	Beta	95% CI	P value	*P*_Intercept_
ApoB	1.11	1.06 to 1.17	<0.001	1.15	1.08 to 1.21	<0.001	1.07	1.02 to 1.12	<0.001	1.18	1.10 to 1.27	<0.001	0.022	1.20	1.11 to 1.30	<0.001	0.112	1.14	1.07 to 1.21	<0.001	0.013
TC	0.91	0.83 to 0.98	<0.001	0.94	0.87 to 1.01	<0.001	0.89	0.82 to 0.97	<0.001	0.87	0.76 to 0.98	<0.001	0.449	0.92	0.82 to 1.02	<0.001	0.571	0.85	0.74 to 0.96	<0.001	0.325
HbA1c	0.00	−0.09 to 0.09	0.979	−0.02	−0.13 to 0.09	0.738	0.01	−0.07 to 0.10	0.791	−0.07	−0.21 to 0.08	0.358	0.236	−0.09	−0.25 to 0.07	0.257	0.214	−0.04	−0.17 to 0.09	0.536	0.310
Calcium	0.04	−0.03 to 0.11	0.230	0.03	−0.05 to 0.12	0.483	0.04	−0.01 to 0.10	0.133	−0.08	−0.17 to 0.02	0.126	0.002	−0.10	−0.22 to 0.02	0.089	0.002	−0.05	−0.14 to 0.03	0.229	0.004
SHBG	0.04	−0.06 to 0.13	0.444	0.02	−0.11 to 0.15	0.750	0.05	−0.05 to 0.14	0.338	0.03	−0.11 to 0.18	0.652	0.958	−0.01	−0.20 to 0.17	0.886	0.619	0.06	−0.09 to 0.21	0.435	0.817
BMR	−0.04	−0.07 to −0.02	0.003	−0.07	−0.12 to −0.02	0.004	−0.06	−0.11 to −0.02	0.009	−0.06	−0.10 to −0.01	0.018	0.552	−0.07	−0.14 to 0.00	0.054	0.949	−0.09	−0.16 to −0.01	0.020	0.385
MPV	0.01	−0.06 to 0.08	0.782	0.00	−0.08 to 0.08	0.974	0.01	−0.06 to 0.08	0.779	0.04	−0.08 to 0.15	0.541	0.570	−0.01	−0.12 to 0.11	0.930	0.880	0.05	−0.06 to 0.17	0.361	0.341
Platelet count	−0.06	−0.17 to 0.04	0.247	−0.07	−0.19 to 0.05	0.262	−0.06	−0.16 to 0.05	0.290	−0.07	−0.24 to 0.10	0.405	0.907	−0.05	−0.23 to 0.13	0.574	0.780	−0.08	0.24 to 0.09	0.372	0.768
Platelet crit	−0.07	−0.18 to 0.04	0.234	−0.08	−0.20 to 0.04	0.202	−0.06	−0.17 to 0.05	0.298	−0.06	−0.23 to 0.11	0.485	0.932	−0.06	−0.24 to 0.12	0.493	0.798	−0.05	−0.23 to 0.12	0.538	0.961
BMI	−0.04	−0.08 to −0.01	0.016	−0.04	−0.08 to 0.00	0.047	−0.05	−0.08 to −0.01	0.009	−0.07	−0.13 to −0.02	0.006	0.122	−0.08	−0.14 to −0.02	0.011	0.099	−0.06	−0.11 to −0.01	0.016	0.406
Weight	−0.06	−0.09 to −0.02	0.002	−0.06	−0.11 to −0.02	0.004	−0.07	−0.12 to −0.03	0.002	−0.07	−0.13 to −0.02	0.012	0.456	−0.09	−0.16 to −0.03	0.007	0.248	−0.07	−0.14 to −0.01	0.027	0.924
Hip circumference	−0.04	−0.09 to 0.00	0.030	−0.05	−0.09 to −0.01	0.017	−0.04	−0.08 to 0.01	0.102	−0.05	−0.12 to 0.01	0.101	0.747	−0.07	−0.13 to 0.00	0.040	0.475	−0.03	−0.1 to 0.03	0.321	0.867
Waist circumference	−0.05	−0.07 to −0.02	0.001	−0.05	−0.08 to −0.01	0.009	−0.06	−0.09 to −0.02	0.002	−0.08	−0.12 to −0.04	<0.001	0.038	−0.10	−0.15 to −0.04	<0.001	0.016	−0.07	−0.12 to −0.02	0.010	0.535

### Replication

Most of the associations for rs12916 replicated using different GWAS studies (Additional file 1: Table S10), where available, with the same direction of associations as the primary analysis. Most replication results had similar effect sizes to the primary ones, particularly for rs12916 T reducing ApoB, LDL-c, total cholesterol, calcium, platelet count, and platelet crit, and for the positive associations of rs12916 with HbA1c, MPV, BMI, hip circumference, and waist circumference. However, sex-specific GWAS for BMR and most body composition traits is currently not available in other publicly available consortia.

## Discussion

Consistent with previous findings about the effects of statins [13], the rs12916 T allele was associated with lower LDL-c, total cholesterol, and ApoB; higher HbA1c [33]; and greater adiposity [12, 34, 35], particularly higher BMI, with little difference by sex. Our study adds by assessing a wider range of phenotypes sex-specifically. We found the rs12916 T allele was associated with higher BMR, and with platelet attributes in both sexes and with lower SHGB and calcium particularly in women, which did not extend to genetically mimicking the major lipid modifiers in current use and largely were not a consequence of LDL-c.

Few RCTs have assessed the effects of statins on BMR, SHBG, or serum calcium, but statins reducing SHBG in women have been previously reported [36, 37]. Previous MR studies have suggested that calcium increases the risk of ischemic heart disease (IHD) [38–40] and SHBG reduces it [40], so these mechanisms together might have a relatively neutral effect on IHD in women. Sex-specific effects of calcium and SHBG on IHD have not been fully assessed, so how these effects would affect specifically women is unknown, although broadly statins appear to have the same effects on IHD in men and women after accounting for testosterone [8]. SHBG inactivates sex hormones, so lowering SHBG might increase the availability of sex hormones and increase the risk of any related conditions. A recent MR study showed that lower SHBG increases the risk of estrogen-positive breast cancer [41]. Effects of SHBG on other cancers in women have not been systematically examined. Concerns were raised about the possibility of statins increasing the risk of breast cancer in women more than a quarter of century ago [42], but are not feasible to investigate in trials. However, a recent MR study did not suggest that genetically mimicking statins increases breast cancer risk [9]. An MR study has suggested that higher BMR increases the risk of colorectal cancer [43]. Recent MR studies have shown that genetically mimicking statins reduces cancer overall, but did not provide sex-specific estimates [9]. Correspondingly, genetically mimicking effects of statins reduced epithelial ovarian cancer [10]. The role of lowering calcium in cancer is unclear and has not been extensively examined.

Few RCTs have examined the effects of statins on platelet attributes. An RCT indicated that statins might reduce platelet count [44], consistent with our findings. We also found that rs12916 T reduced platelet crit, and increased MPV. A cohort study found platelet count was positively associated with CVD risk and mortality [45], as did an MR study [46]. Few RCTs have examined these questions, but MPV is increasingly realized to be important to CVD [47, 48], and platelet crit may be associated with stroke [49], so these may be additional mechanisms by which protective effects of statins are actuated.

Statins increasing BMI [12, 34] and HbA1c [33, 50] have been reported previously, consistent with our findings. We also found that the rs12916 T allele affected BMR, fat-mass, and fat-free mass. Genetically mimicked PCSK9 inhibitors and ezetimibe were not associated with BMI or BMR, and genetically mimicked PCSK9 inhibitors were not associated with HbA1c, consistent with previous findings [51]. LDL-c had minor effects on BMR, anthropometrics, and most body composition traits. Given statins adversely impact body composition and glycemic traits more strongly than other lipid modifiers but have similar effects on IHD per unit change in LDL-c, it suggests that statins have greater effects via lipid and/or non-lipid mechanisms than other lipid modifiers.

Differences in pleiotropic effects of genetically mimicked statins compared to PCSK9 inhibitors and ezetimibe may be related to the differences of their mechanisms of action. PCSK9 inhibitors reduce the degradation of LDL receptors by blocking PCSK9 [52], resulting in lower levels of circulating LDL-c [53]. Ezetimibe only inhibits the absorption of cholesterol [54]. Statins target cholesterol synthesis [55], including de novo synthesis in Leydig cells, so statins specifically affect hormones, although the mechanism by which statins might affect SHBG in women is less clear.

This study aimed to identify pleiotropic effects of statins, and whether they might be mediated by the target of statins, LDL-c, by testing the associations of LDL-c with any pleiotropic effects. We found the pleiotropic effects of statins did not appear to be driven by LDL-c, and so are specific effects of statins. Exactly, what drives these pleiotropic effects of statins has not been definitively established, nor has their interrelationships, which may well be complex. A possibility is that the pleiotropic effects of statins are driven by effects of statins on BMI. However, statins raise BMI in both sexes (Table 1), while genetically mimicked effects on calcium and SHBG were specific to women, suggesting a more complex explanation. A trial of gastric bypass suggested that BMI increases platelet count [56] but genetically predicted BMI does not appear to affect platelet count in women (Additional file 1: Table S14). However, BMI is well-known to play a crucial role in BMR in both sexes [57]. Using multivariable MR, the pleiotropic effects of genetically mimicking statins on BMR were not independent of BMI (Additional file 1: Table S15), suggesting that any effects of statins on BMR are due to statins raising BMI.

Despite a comprehensive sex-specific scan in the largest available studies, this study has some limitations. First, not all phenotypes of interest were available for the main analysis or for replication, such as very-low-density lipoprotein and some other lipid sub-fractions, and some body composition traits; however, effects of statins on lipid fractions have been examined before [13–15]. Second, this study is systematic and comprehensive but is also agnostic. As such, it uses a stringent test for significance to avoid chance findings, so may not replicate all known effects of statins. Instead, this study may provide information about unknown or overlooked potential effects of statins, which is important because of the very widespread use of statins globally. The multiple comparison cut-off using a Bonferroni correction is suitable for agnostic studies, as here. An agnostic study design is most appropriate for identifying pleiotropic effects which have not been considered before rather than for replicating findings based on known physiological pathways, which can be evaluated on different criteria in the context of all the other evidence. The largest available GWAS of IHD does not show rs12916 associated with IHD at genome-wide significance [58], which does not invalidate the GWAS or the role of statins in preventing IHD; instead, the GWAS provides information about other, possibly overlooked, factors that could be relevant to IHD. Similarly, here, this study does not invalidate known relations of statins with IHD or testosterone, which have been demonstrated in a meta-analysis of RCTs [59, 60], but provides additional insight about potentially relevant pleiotropic effects of statins. Overall, this study design is most appropriate for identifying pleiotropic effects of statins rather than replicating known effects. We cannot exclude the possibility that some novel effects of statins have been missed, which could be addressed by repeating this study when larger sex-specific genetic studies are available. Third, we used rs12916 and associated genetic variants as a surrogate for the pharmacological effects of statins [12], which mimics a life-long small dose of endogenous statins [8, 10, 13], so the MR estimates represent life-long inhibition of HMGCR [8, 10] and do not necessarily reflect the effects of statin treatment which generally starts in middle age [10, 61]. These estimates are usually different in magnitude from the short-term effects of pharmacologic interventions in an RCT [62] although similar effect sizes have been seen for genetically mimicked statins and use of statins [13]. Fourth, the UK Biobank is not representative of the UK population. However, no confounding and no selection bias are the criteria for an internally valid study of associations, not population representativeness [63]. The UK Biobank has shown similar associations to an equivalent population-representative study [64]. However, as with any study recruited in middle to older age, the UK Biobank is missing people who died before recruitment from their genetic make-up, from a condition of interest, or from a competing risk of such a condition, which may generate selection bias particularly for conditions that share etiology with diseases that cause death before recruitment [65], so effects of statins on late-onset diseases may have been missed. The UK Biobank study undoubtedly comprises healthy volunteers, less vulnerable to disease, which may bias associations with disease towards null. Fifth, the underlying studies mostly concern populations of European descent, due to data availability, which may limit the generalizability of these findings to other populations. It would be extremely beneficial to validate these findings in consortia more representative of the global population.

## Conclusion

After systematic examination, we found that genetically mimicking statins was similarly associated with lower lipids as other major lipid modifiers and was positively associated with adiposity and HbA1c as expected. We also found genetically mimicking statins was associated with higher BMR, consistent with effects on adiposity. In addition, genetically mimicking statins was also associated with lower serum calcium and SHBG and was related to platelet attributes. Associations with SHBG and calcium were specific to women. Besides lipid modulation, inferred from the genetic evidence obtained, lower platelet count may contribute to the benefits of statins, whereas serum calcium could be in a potential pathway that contributes to the benefits of statins on CVD in specifically women. The consequences of statins possibly lowering SHBG on hormone-related conditions in women require further investigation, initially using study designs, such as Mendelian randomization which can provide high-quality evidence expeditiously without any risk. Such information could identify whether statins’ effects on SHBG in women are likely to make any material difference to the net benefits of statins, so as to further inform the level of future risk at which statin use should be initiated in women for primary prevention.

## Supplementary Information

**Additional file 1: Table S1.** Established genetic variants in the *HMGCR*, *PCSK9* and *NCP1L1* gene that mimic statins, PCSK9 inhibitors and ezetimibe respectively]. **Table S2.** Correlation matrix for genetic variants mimicking statins. **Table S3.** Correlation matrix for genetic variants mimicking PCSK9 inhibitors. **Table S4.** Correlation matrix for genetic variants mimicking ezetimibe. **Table S5.** 56 established independent SNPs genetically predicting LDL-c. **Table S6.** PheWAS table of rs12916. **Table S7.** Number of significant associations of rs12916 with phenotypes by category. **Table S8.** Sex-specific associations of genetically mimicked statins, PCSK9 inhibitors and ezetimibe (based on rs12916, rs11591147 and rs10260606, respectively) with body composition traits. **Table S9.** Sex-specific univariable MR of genetically predicted LDL-c on binary traits and body composition traits. **Table S10.** Replication table. **Table S11.** Sensitivity analysis for genetically mimicking statins. **Table S12.** Sensitivity analysis for genetically mimicking PCSK9 inhibitors. **Table S13.** Sensitivity analysis for genetically mimicking ezetimibe. **Table S14.** Univariable MR results of BMI on platelet count and BMR. **Table S15.** Multivariable MR results of genetically mimicked statins on BMR adjusting for BMI.

**Additional file 2: Figure I.** Manhattan plots for rs12916 with all phenotypes included by category. The x-axis shows the phenotypes by category, and the y-axis shows the -log_10_ transformed *P* values. The blue line indicates the corrected statistical significance level, *P* = 2.9 × 10^-5^. The categories of phenotypes (from left to right on the x-axis) are blood biochemistry, circulatory, blood count, digestive, endocrine, neoplasms, respiratory, symptoms, physical measures, other categories (continuous) and other categories (binary). Other categories (continuous) include gynaecologic and obstetric, mental health, sense organs and others. Other categories (binary) include neurological, musculoskeletal, gynaecologic and obstetric, hematopoietic, dermatologic, genitourinary, mental health, infectious diseases, sense organs, injuries and poisonings and others. **Figure II.** Comparison between primary and sensitivity analysis for genetically mimicking statins. **Figure III.** Comparison between primary and sensitivity analysis for genetically mimicking PCSK9 inhibitors. **Figure IV.** Comparison between primary and sensitivity analysis for genetically mimicking ezetimibe.

## Data Availability

The datasets generated and analyzed during the current study are available in the UK Biobank repository with sex-specific datasets available, [https://docs.google.com/spreadsheets/d/1kvPoupSzsSFBNSztMzl04xMoSC3Kcx3CrjVf4yBmESU/edit?ts=5b5f17db#gid=178908679], and the TwoSampleMR R package, [https://github.com/MRCIEU/TwoSampleMR].

## Abbreviations

ApoB: Apolipoprotein B
BMI: Body mass index
BMR: Basal metabolic rate
CARDIoGRAMplusC4D: Coronary Artery Disease Genome wide Replication and Meta-analysis plus The Coronary Artery Disease Genetics
CETP: Cholesteryl ester transfer protein
CVD: Cardiovascular disease
GLGC: The Global Lipids Genetics Consortium
GWAS: Genome-wide association studies
HbA1c: Glycated hemoglobin
ICD: International Classification of Diseases
IEU: Integrative Epidemiology Unit
IHD: Ischemic heart disease
IVW: Inverse-variance weighting
LDL-c: Low-density lipoprotein cholesterol
MPV: Mean platelet volume
MR: Mendelian randomization
OR: Odds ratio
PCSK9: Proprotein convertase subtilisin/kexin type 9
PheWAS: Phenome-wide association study
SD: Standard deviation
SHBG: Sex hormone-binding globulin
SNP: Single nucleotide polymorphism
TC: Total cholesterol
