# Supplementary material for: A phenome-wide association study of genetically mimicked statins
Source: BMC Med. 2021 Jun 30;19:151. doi: 10.1186/s12916-021-02013-5 (PMC8244164; doi:10.1186/s12916-021-02013-5)
Supplement: Supplementary file 2 — Additional file 2: Figure I. Manhattan plots for rs12916 with all phenotypes included by category. The x-axis shows the phenotypes by category, and the y-axis shows the -log10 transformed P values. The blue line indicates the corrected statistical significance level, P = 2.9 × 10^-5^. The categories of phenotypes (from left to right on the x-axis) are blood biochemistry, circulatory, blood count, digestive, endocrine, neoplasms, respiratory, symptoms, physical measures, other categories (continuous) and other categories (binary). Other categories (continuous) include gynaecologic and obstetric, mental health, sense organs and others. Other categories (binary) include neurological, musculoskeletal, gynaecologic and obstetric, hematopoietic, dermatologic, genitourinary, mental health, infectious diseases, sense organs, injuries and poisonings and others. Figure II. Comparison between primary and sensitivity analysis for genetically mimicking statins. Figure III. Comparison between primary and sensitivity analysis for genetically mimicking PCSK9 inhibitors. Figure IV. Comparison between primary and sensitivity analysis for genetically mimicking ezetimibe. [file 12916_2021_2013_MOESM2_ESM.pdf]

Figure I

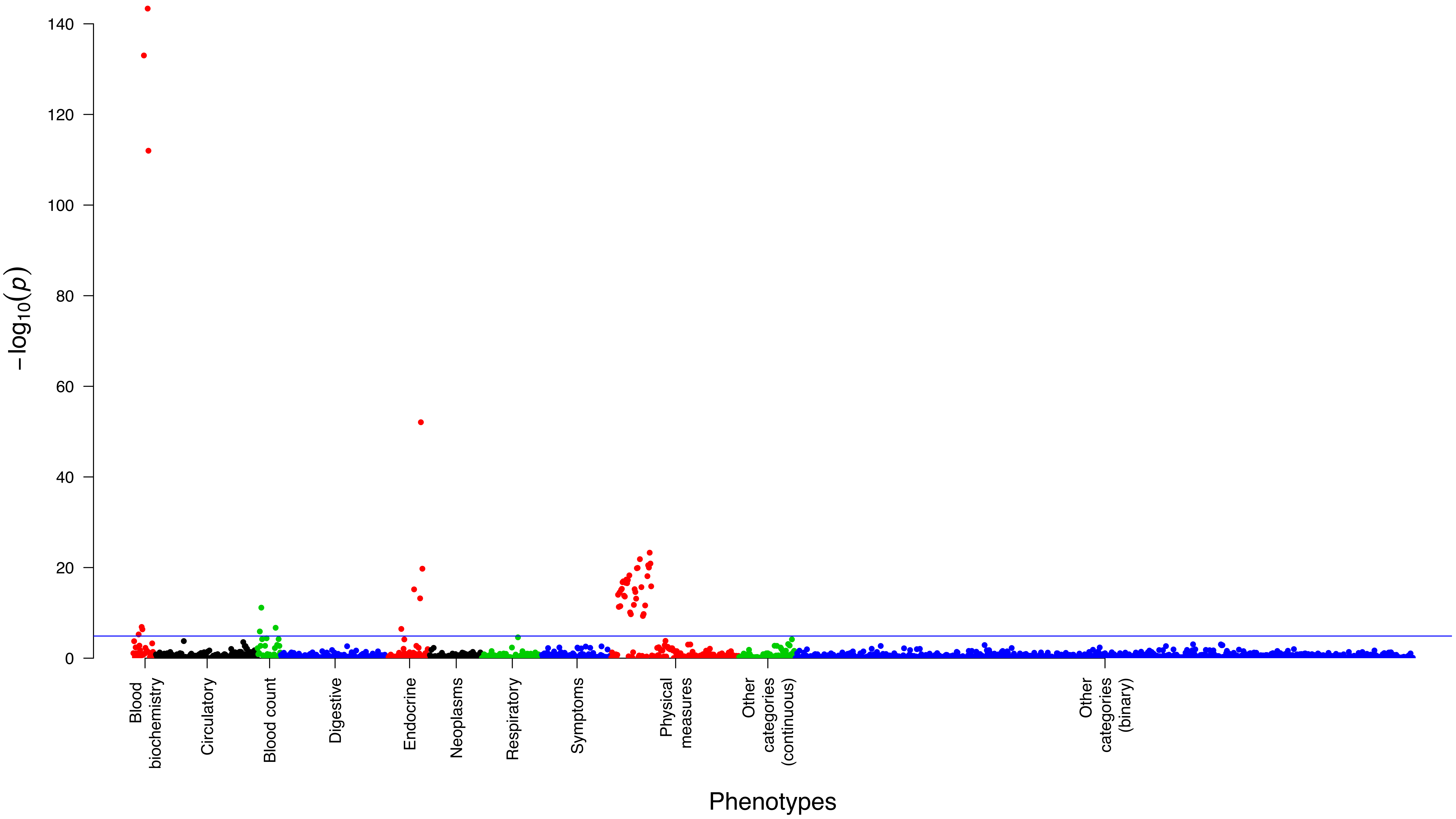

$-\log_{10}(p)$
140
120
100
80
60
40
20
0
Blood biochemistry
Circulatory
Blood count
Digestive
Endocrine
Neoplasms
Respiratory
Symptoms
Physical measures
Other categories (continuous)
Other categories (binary)
Phenotypes

Figure II

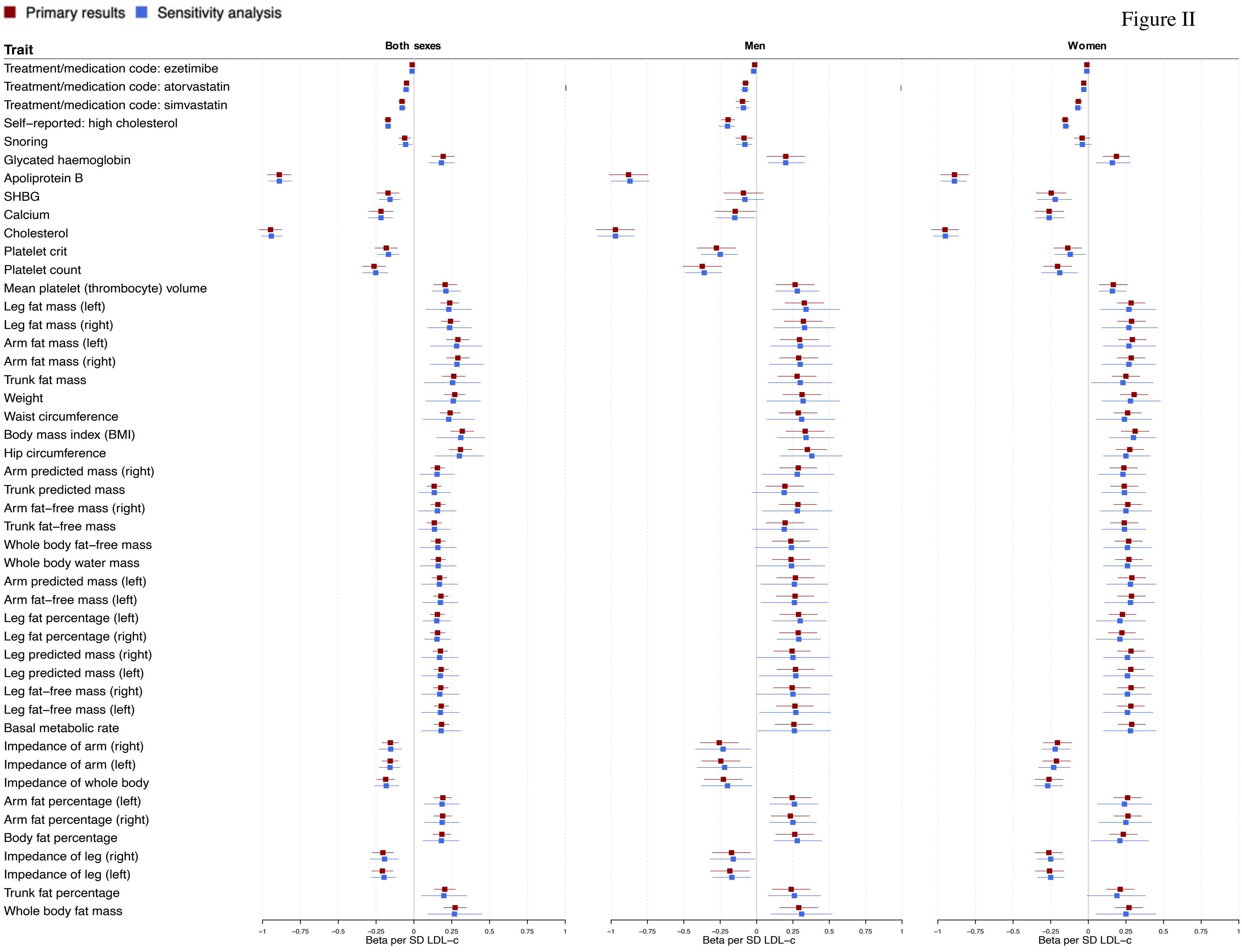

Primary results
Sensitivity analysis
Trait
Both sexes
Men
Women
Treatment/medication code: ezetimibe
Treatment/medication code: atorvastatin
Treatment/medication code: simvastatin
Self−reported: high cholesterol
Snoring
Glycated haemoglobin
Apoliprotein B
SHBG
Calcium
Cholesterol
Platelet crit
Platelet count
Mean platelet (thrombocyte) volume
Leg fat mass (left)
Leg fat mass (right)
Arm fat mass (left)
Arm fat mass (right)
Trunk fat mass
Weight
Waist circumference
Body mass index (BMI)
Hip circumference
Arm predicted mass (right)
Trunk predicted mass
Arm fat−free mass (right)
Trunk fat−free mass
Whole body fat−free mass
Whole body water mass
Arm predicted mass (left)
Arm fat−free mass (left)
Leg fat percentage (left)
Leg fat percentage (right)
Leg predicted mass (right)
Leg predicted mass (left)
Leg fat−free mass (right)
Leg fat−free mass (left)
Basal metabolic rate
Impedance of arm (right)
Impedance of arm (left)
Impedance of whole body
Arm fat percentage (left)
Arm fat percentage (right)
Body fat percentage
Impedance of leg (right)
Impedance of leg (left)
Trunk fat percentage
Whole body fat mass
−1 −0.75 −0.5 −0.25 0 0.25 0.5 0.75 1
Beta per SD LDL−c
Beta per SD LDL−c
Beta per SD LDL−c

Figure III

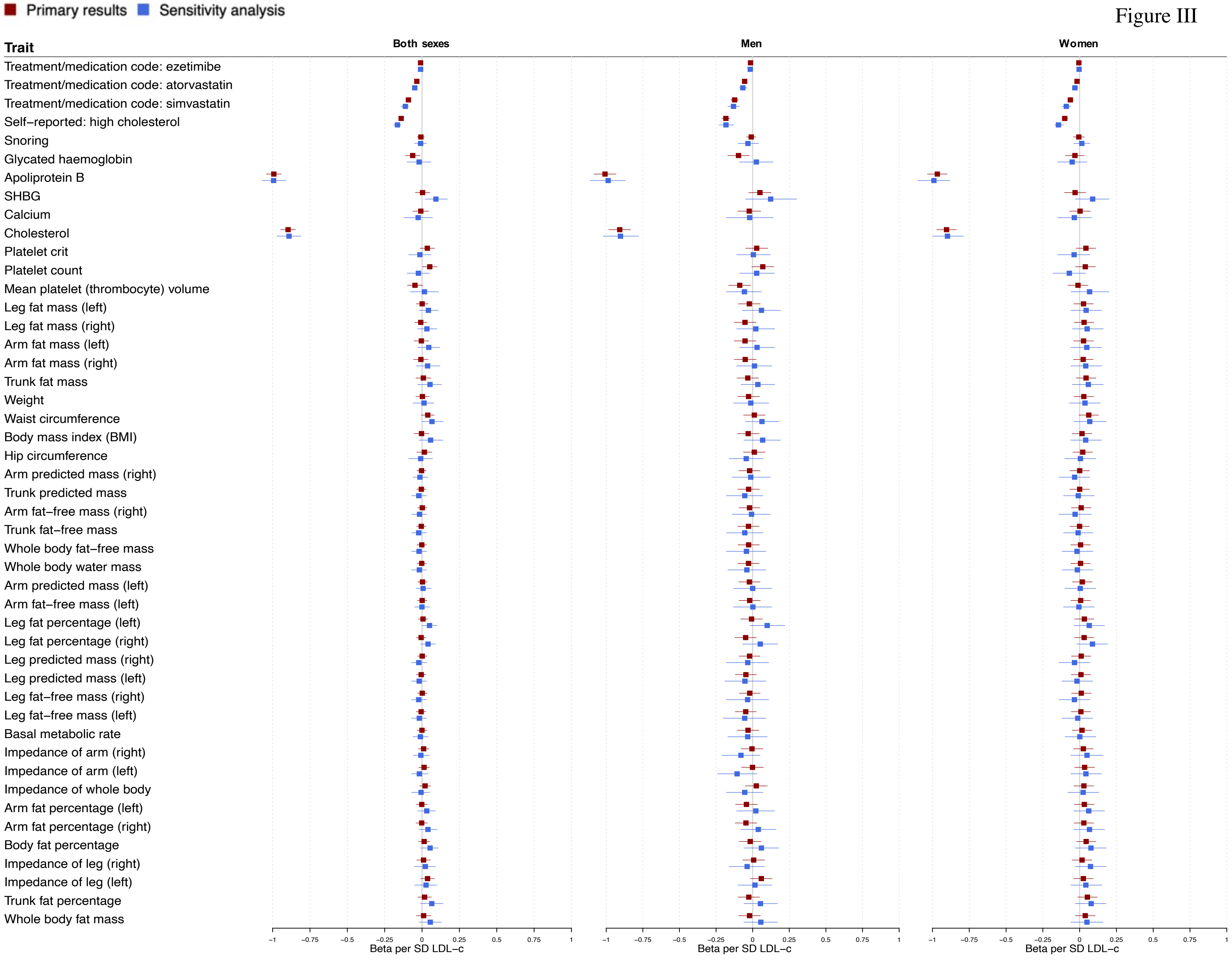
Primary results
Sensitivity analysis
Trait
Both sexes
Men
Women
Treatment/medication code: ezetimibe
Treatment/medication code: atorvastatin
Treatment/medication code: simvastatin
Self−reported: high cholesterol
Snoring
Glycated haemoglobin
Apoliprotein B
SHBG
Calcium
Cholesterol
Platelet crit
Platelet count
Mean platelet (thrombocyte) volume
Leg fat mass (left)
Leg fat mass (right)
Arm fat mass (left)
Arm fat mass (right)
Trunk fat mass
Weight
Waist circumference
Body mass index (BMI)
Hip circumference
Arm predicted mass (right)
Trunk predicted mass
Arm fat−free mass (right)
Trunk fat−free mass
Whole body fat−free mass
Whole body water mass
Arm predicted mass (left)
Arm fat−free mass (left)
Leg fat percentage (left)
Leg fat percentage (right)
Leg predicted mass (right)
Leg predicted mass (left)
Leg fat−free mass (right)
Leg fat−free mass (left)
Basal metabolic rate
Impedance of arm (right)
Impedance of arm (left)
Impedance of whole body
Arm fat percentage (left)
Arm fat percentage (right)
Body fat percentage
Impedance of leg (right)
Impedance of leg (left)
Trunk fat percentage
Whole body fat mass
−1
−0.75
−0.5
−0.25
0
0.25
0.5
0.75
1
Beta per SD LDL−c

Figure IV

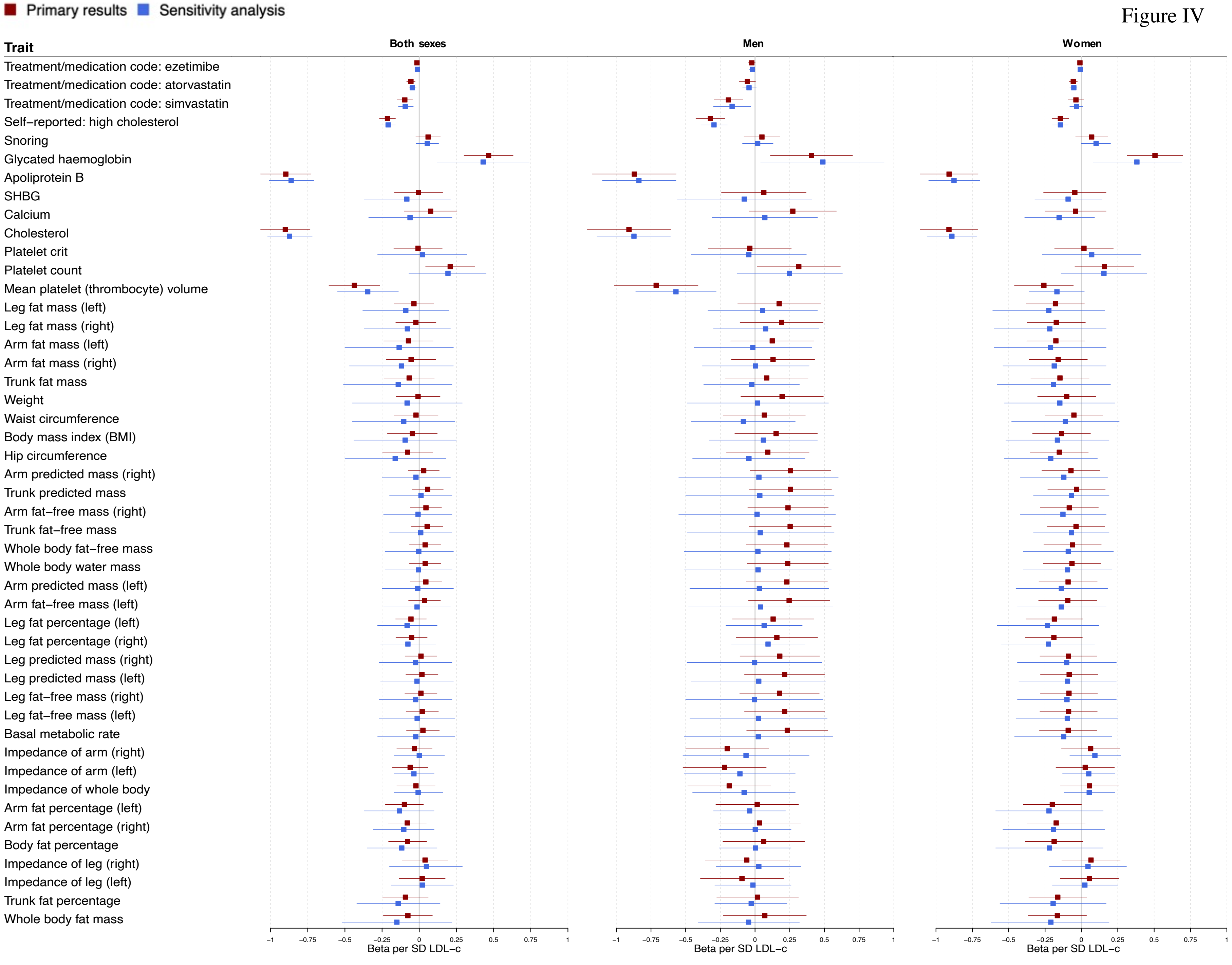
Primary results
Sensitivity analysis
Trait
Both sexes
Men
Women
Treatment/medication code: ezetimibe
Treatment/medication code: atorvastatin
Treatment/medication code: simvastatin
Self−reported: high cholesterol
Snoring
Glycated haemoglobin
Apoliprotein B
SHBG
Calcium
Cholesterol
Platelet crit
Platelet count
Mean platelet (thrombocyte) volume
Leg fat mass (left)
Leg fat mass (right)
Arm fat mass (left)
Arm fat mass (right)
Trunk fat mass
Weight
Waist circumference
Body mass index (BMI)
Hip circumference
Arm predicted mass (right)
Trunk predicted mass
Arm fat−free mass (right)
Trunk fat−free mass
Whole body fat−free mass
Whole body water mass
Arm predicted mass (left)
Arm fat−free mass (left)
Leg fat percentage (left)
Leg fat percentage (right)
Leg predicted mass (right)
Leg predicted mass (left)
Leg fat−free mass (right)
Leg fat−free mass (left)
Basal metabolic rate
Impedance of arm (right)
Impedance of arm (left)
Impedance of whole body
Arm fat percentage (left)
Arm fat percentage (right)
Body fat percentage
Impedance of leg (right)
Impedance of leg (left)
Trunk fat percentage
Whole body fat mass
−1 −0.75 −0.5 −0.25 0 0.25 0.5 0.75 1
Beta per SD LDL−c
